# Temporal Artery Biopsy for Diagnosing Giant Cell Arteritis: A Ten-year Review

**DOI:** 10.18502/jovr.v15i2.6738

**Published:** 2020-04-06

**Authors:** Kaveh Abri Aghdam, Mostafa Soltan Sanjari, Navid Manafi, Shabnam Khorramdel, Sayyed Amirpooya Alemzadeh, Roshanak Ali Akbar Navahi

**Affiliations:** Eye Research Center, The Five Senses Institute, Rassoul Akram Hospital, Iran University of Medical Sciences, Tehran, Iran

**Keywords:** Anterior Ischemic Optic Neuropathy, Giant Cell Arteritis, Temporal Arteritis, Temporal Artery Biopsy

## Abstract

**Purpose:**

To assess the use of temporal artery biopsy (TAB) in diagnosing giant cell arteritis (GCA) and to evaluate patients' clinical and laboratory characteristics.

**Methods:**

We conducted a retrospective chart review of patients with suspected GCA who underwent TAB and had complete workup in a tertiary center in Iran between 2008 and 2017. The 2016 American College of Rheumatology (ACR) revised criteria for early diagnosis of GCA were used for each patient for inclusion in this study.

**Results:**

The mean age of the 114 patients in this study was 65.54 ± 10.17 years. The mean overall score according to the 2016 ACR revised criteria was 4.17 ± 1.39, with 5.82 ± 1.28 for positive biopsies and 3.88 ± 1.19 for negative biopsies (p <0.001). Seventeen patients (14.9%) had a positive biopsy. Although the mean post-fixation specimen length in the biopsy-positive group (18.35 ± 6.9 mm) was longer than that in the biopsy-negative group (15.62 ± 8.4 mm), the difference was not statistically significant (*P* = 0.21). There was no statistically significant difference between the groups in terms of sex, serum hemoglobin, platelet count, and erythrocyte sedimentation rate. There were statistically significant differences between the biopsy-negative and biopsy-positive groups with respect to patients' age and C-reactive protein level (*P* < 001 and *P* = 0.012, respectively).

**Conclusion:**

The majority of TABs were negative. Reducing the number of redundant biopsies is necessary to decrease workload and use of medical services. We suggest that the diagnosis of GCA should be dependent on clinical suspicion.

##  INTRODUCTION

Giant cell arteritis (GCA) is a systemic immune-related vasculitis involving medium- and large-sized arteries, especially of the carotid branches, including the superficial temporal, ophthalmic, posterior ciliary, and occipital arteries.^[1,2]^ The inflammation of the arterial wall causes luminal obstruction and tissue ischemia.^[3]^ The involvement of these vessels can cause visual impairment or loss. Vision loss in GCA is usually due to arteritic anterior ischemic optic neuropathy, which is optic nerve head ischemia caused by inflammation of the wall of the posterior ciliary arteries.^[1]^

Correct diagnosis and urgent treatment with systemic corticosteroids are necessary to prevent major ischemic complications.^[4]^ Although there is no single test for diagnosing GCA, the American College of Rheumatology (ACR) 1990 GCA classification criteria can assist in the diagnosis of suspected cases.^[5]^ The presence of at least three of these five criteria carries a sensitivity of 93.5% and a specificity of 91.2% for GCA.^[5,6]^ In 2016, a new and more extensive set of criteria for early diagnosis of the GCA was proposed.^[7]^ Table 1 shows the 2016 revised ACR (rACR) criteria for early diagnosis of GCA (previously called temporal arteritis). It has been suggested that in the presence of at least 3 out of 11 points, with at least one point belonging to domain I, a diagnosis of GCA can be made.

**Table 1:** The 2016 revised ACR criteria for the diagnosis of GCA ${}^{a}$


**Score**	**Entry**
N/A	Age at onset ≥ 50 years
	Absence of exclusion criteria ${}^{b}$
Domain I	
1	New-onset localized headache, ${}^{c}$ 1 point (p)
1	Sudden onset of visual disturbance, ${}^{c}$ 1 point
2	Polymyalgia rheumatica, 2 points
1	Jaw claudication, ${}^{c}$ 1 point
2	Abnormal temporal artery, ${}^{d}$ up to 2 points
Domain II	
1	Unexplained fever and/or anemia, 1 point
1	ESR ≥ 50 mm/hour, ${}^{e}$ 1 point
2	Compatible pathology, ${}^{f}$ up to 2 points
ACR, American College of Rheumatology; ESR, erythrocyte sedimentation rate; GCA, giant cell arteritis; N/A, not applicable ${}^{a}$ In the presence of three points or more out of the eleven with at least one point belonging to domain I along with all entry criteria, a diagnosis of GCA can be established. ${}^{b}$ The exclusion criteria included ear, nose, and throat or/and eye inflammation; kidney, skin, or peripheral nervous system involvement; lung infiltration; lymphadenopathies; stiff neck; and digital gangrene or ulceration. ${}^{c}$ No other etiologies can better explain any one of the criteria. ${}^{d}$ Enlarged and/or pulseless temporal artery (one point)/tender temporal artery (one point). ${}^{e}$ It must be ignored in the presence of polymyalgia rheumatica. ${}^{f}$ Vascular and/or perivascular fibrinoid necrosis along with leucocyte infiltration (one point) and granuloma (one point).	

**Table 2:** Comparison between TAB-negative and TAB-positive patients according to the 2016 revised ACR criteria


	**TAB-positive (** ***n*** ** = 17)**	**TAB-negative (** ***n*** ** = 97)**	***P*** **-value**
ESR (mm/hour)	52.50 ± 33.28	52.30 ± 28.21	0.98
Hemoglobin (g/dl)	12.40 ± 1.95	11.97 ± 1.80	0.38
Age (years)	75.66 ± 8.32	63.92 ± 9.51	< 0.001
Temporal tenderness	29.41%	35.05%	0.61
Jaw claudication	41.2%	26.8%	0.45
Pulseless temporal artery	17.64%	10.30%	0.26
ACR, American College of Rheumatology; ESR, erythrocyte sedimentation rate; TAB, temporal artery biopsy			

**Table 3:** Comparison of the first and second five years in terms of biopsy result and length


**Biopsy year**	**Number of biopsies**	**Biopsy result**		***P*** **-value**	**Biopsy length (mm) **	***P*** **-value**
	**Negative**	**Positive**		
First five years	59	53	6	0.06	14.94 ± 9.25	0.03
Second five years	47	36	11	17.42 ± 6.88	

**Table 4:** Previous studies on the length of superficial temporal artery biopsy (STAB)


**Study**	**Year**	**Location**	**Study design**	**Number of cases**	**Number of biopsies**	**Mean biopsy length (mm)**	**Positivity rate (%) ${}^{a}$ **	**Main finding and conclusion**
Allison et al^[23]^	1984	UK	Retrospective review	132	132	7.9	64	Size of 7 mm is recommended for more accurate results
Kent et al^[24]^	1990	USA	Retrospective review	70	73	N/S	11.4	Generous biopsy of ∼ 5 cm of fresh vessel recommended to confirm a suspected diagnosis of temporal arteritis
Achkar et al^[25]^	1994	USA	Consecutive case series	535	535	36.32	33	Suggested to obtain samples ≥ 20 mm
Sudlow et al^[26]^	1997	Scotland	Retrospective review	N/S	200	9.14	27.02	Longer specimens may be more likely to yield a positive result
Taylor-Gjevre et al^[27]^	2005	Canada	Retrospective review	141	141	17.6	27	More positive results at post-fixation length of 10 mm
Arashvand et al^[28]^	2006	UK	Retrospective review	N/S	117	11.95 ± 7.91	26	Raising or lowering the minimum threshold length did not yield a statistically significant difference in the rate of positive results
Mahr et al^[29]^	2006	France	Retrospective review	1520	1520	13.3 ± 7.2	14.7	Biopsy sample size of 5 mm is adequate
Sharma et al^[29]^	2007	Australia	Retrospective observational study	157	157	11.85	N/S	Specimens of ≥ 20 mm were 2.8 times more likely to show features of GCA than those < 20 mm
Breuer et al^[31]^	2009	Israel	Retrospective review	173	305	11.9	35.4	Longer samples are more accurate
Ypsilantis et al^[32]^	2011	UK	Cohort	966	966	10	21.4	A 10-mm sample is satisfactory (post-fixation length of ≥ 7 mm)
Kaptanis et al^[33]^	2014	UK	Retrospective review	149	151	6.4 ± 3	13.3	No relation between length and results; hence post-fixation length of 6 mm is satisfactory (biopsy length > 10 mm)
Au et al^[4]^	2016	Australia	Retrospective observational	96	96	16 ± 7.3	20.8	Length of biopsy is not an independent factor in positivity rate
Grossman et al^[34]^	2016	Israel	Retrospective analysis	240	240	10.7 ± 5.7	25.83	Length of biopsy is not a determining factor
Gajree et al^[35]^	2017	Scotland	Retrospective analysis	715	715	11.64 ± 6.4	35	Length of specimen does not necessarily change the likelihood of a positive biopsy
Papadakis et al^[36]^	2017	Germany	Retrospective analysis	116	116	9.4	55.2	TAB length is not associated with the TAB diagnostic yield in patients with clinical suspicion of GCA
Oh et al^[12]^	2018	Australia	Retrospective case-control of consecutive cohort	538	538	17.6 ± 9.1	23.4	Biopsy length of ≥ 15 mm is suggested
Current study	2019	Iran	Retrospective analysis	114	114	16.05 ± 8.27	14.9	TAB length is not significantly different in positive and negative biopsies; also, the majority of TABs are negative
GCA, giant cell arteritis; N/S, not stated; TAB, temporal artery biopsy ${}^{a}$ Rate of positive biopsies in all biopsies							

Temporal artery biopsy (TAB) is indicated for diagnosing suspected GCA cases.^[8]^ Although the value of TAB has been recently disputed because of its high false-negative rates, it remains the gold standard for diagnosis.^[9]^

The purpose of the current study was to investigate the use of TAB in diagnosing GCA at a tertiary center in Iran over a 10-year period. We investigated and discussed the clinical and laboratory results of patients with TAB-proven GCA and TAB-negative cases according to the ACR criteria.

##  METHODS

We conducted a retrospective chart review in the Neuro-ophthalmology Clinic at the Rassoul Akram Hospital, a tertiary referral center in Tehran, Iran, between 2008 and 2017. The study was approved by the Ethics Committee of the Iran University of Medical Sciences and adhered to the tenets of the Declaration of Helsinki. We reviewed the medical records of patients with suspected GCA, including pathology reports of all TABs within this period. The 2016 rACR criteria for early diagnosis of giant cell (temporal) arteritis were considered for each patient for inclusion in this study. The age-specific maximal normal erythrocyte sedimentation rate (ESR) was calculated according to the following formulas: age in years/2 for men and (age in years +10)/2 for women.^[10]^ Superficial TABs (STABs) were performed under local anesthesia using routine surgical techniques by either ophthalmology, vascular surgery, or neurosurgery residents with a wide spectrum of surgical expertise.^[11]^ The specimens were fixed in 10% formalin. The lengths of the formalin-fixed STAB samples on macroscopic examination were included in the reports. The specimens were processed using standard protocols for embedding tissue in paraffin wax blocks. The specimens were completely embedded, and histopathological sections were cut at three or more levels and examined after hematoxylin and eosin staining. All patients had undergone TAB within 1–2 days after starting steroid treatment.

The checklist information for each patient included age, sex, laboratory test results (hemoglobin, platelet count, ESR, and C-reactive protein [CRP] levels), post-fixation STAB length, histological findings, side of biopsy, and notes of the ophthalmology team. Histological findings were documented with respect to the presence of multinucleated giant cells of Langerhans, inflammatory cell infiltration, intimal proliferation, and fragmentation of the internal elastic lamina. Details on the exact number of sections and levels examined were documented in most reports. Some of the cases were excluded for having an inappropriate biopsy specimen. All patients in the TAB-positive group had leukocyte infiltration (one point based on the rACR) and granuloma (one point based on the rACR) in their histopathology specimens.

### Statistical Analysis

SPSS software version 22.0 (IBM Corp., Armonk, NY) was used for statistical analyses. The Student's *t*-test was used for analyzing quantitative variables, and the Chi square test was used for qualitative variables. Results are reported as mean ± standard deviation. *P* values < 0.05 were considered statistically significant. Statistical analysis was also performed to compare the first and second five years due to variation in specialties performing the biopsies and differences in the TAB specimen length over 10 years.

##  RESULTS

**Figure 1:**
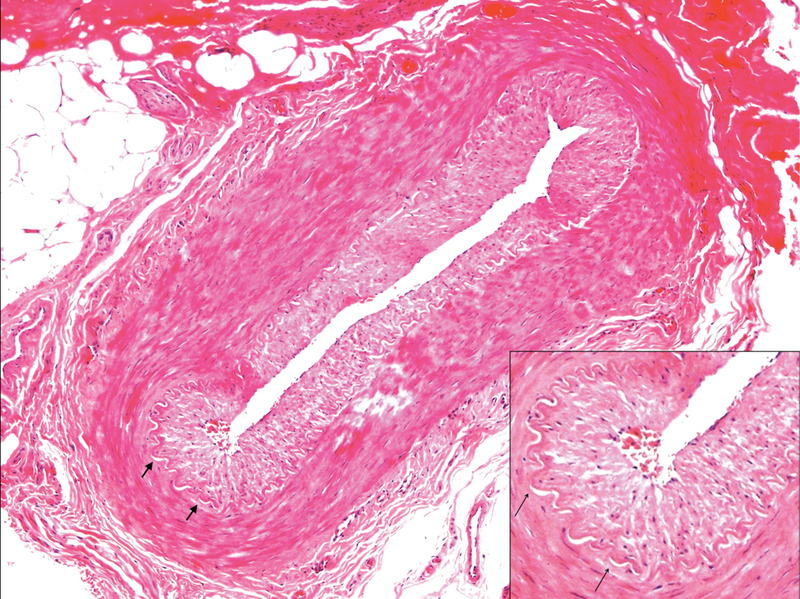
Histopathology section of a negative temporal artery biopsy. Note the intact lamina elasticum (short arrows) and the absence of inflammation (hematoxylin and eosin staining, 100 × magnification).

**Figure 2:**
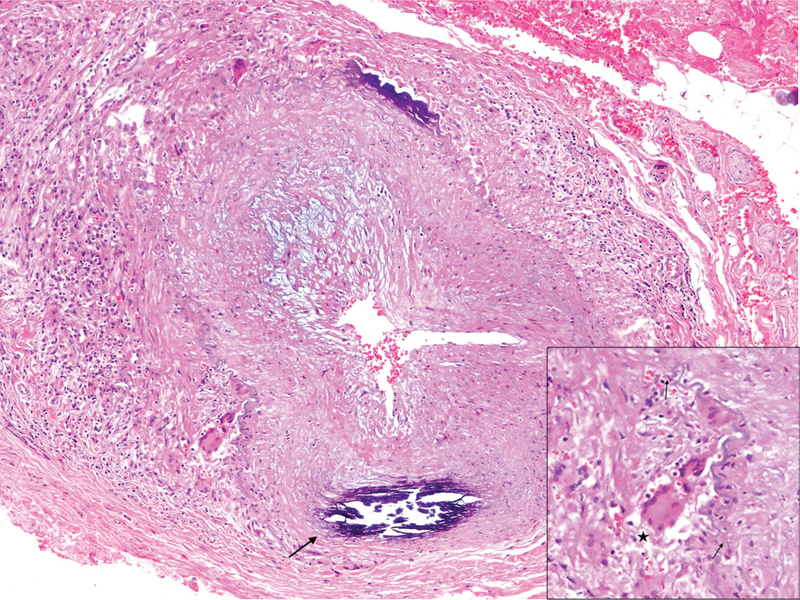
GCA-positive temporal artery biopsy (H&E staining, 100 × magnification). Note the narrowing of the arterial lumen and infiltration of inflammatory cells and multinucleated giant cells (asterisk) in addition to the destruction of the lamina elasticum (short arrows). Age-related intramural calcification is also noted in this specimen (long arrow). GCA, giant cell arteritis; H&E, hematoxylin and eosin.

In the primary collection of patient records, 257 patient charts were reviewed and analyzed. The patients who lacked the 2016 rACR criteria, an appropriate biopsy report, or the associated blood test results were excluded. After also excluding patients with incomplete medical records, 114 patients with complete required information in their charts who met the 2016 rACR criteria were evaluated. Of these, 43 patients (37.7%) were male and 71 (62.3%) were female, with an overall mean age of 65.54 ± 10.17 years at the time of biopsy. Of the 114 biopsies included in the study, 17 (14.9%) showed the characteristics of GCA. The mean age of TAB-negative patients [Figure 1] was 63.92 ± 9.51 years compared to a mean age of 75.66 ± 8.32 years in TAB-positive cases [Figure 2] (*P* < 0.001). In patients with positive biopsies, the mean CRP level was 30.50 ± 32.35 mg/L, whereas in those with negative biopsies, it was 13.68 ± 18.91 mg/L (*P* = 0.009). An abnormal ESR and ESR > 50 mm/hour were present in 61% and 44% of cases, respectively. The mean overall score according to the 2016 ACR revised criteria was 4.17 ± 1.39, with 5.82 ± 1.28 for positive biopsies and 3.88 ± 1.19 for negative biopsies (p < 0.001).

Although the mean post-fixation specimen length in the biopsy-positive group (18.35 ± 6.9 mm) was longer than that in the biopsy-negative group (15.62 ± 8.4 mm), the difference was not statistically significant (*P* = 0.21). Of the 114 patients, 96 cases presented with acute visual loss; 83 (86.4%) of them had anterior ischemic optic neuropathy (69 subjects in the TAB-negative group and 14 in the TAB-positive group); 8 (8.3%) were diagnosed with posterior ischemic optic neuropathy (seven cases in the TAB-negative group and one in the TAB-positive group); and 5 (5.2%) cases had central retinal artery occlusion (four cases in the TAB-negative group and one in the TAB-positive group). Three other patients developed acute cranial nerve palsies; two patients had sudden third nerve palsy, and one case presented with acute sixth nerve palsy.

Table 2 compares the TAB-positive and TAB-negative groups in terms of 2016 rACR scoring. Except for age, there was no significant difference between the biopsy-positive and biopsy-negative groups in terms of their symptoms (all *P*-values > 0.05).

We also compared the results of first and second five years to evaluate the human factor effect during this time period [Table 3]. We observed a significant increase in the specimen length obtained during the second five years (*P* = 0.03) but nonsignificant rise in the number of positive biopsies (odds ratio = 2.69, *P* = 0.06).

##  DISCUSSION

This study describes the results of a cohort of 114 patients who met the 2016 rACR criteria for the diagnosis of GCA and underwent TAB over a 10-year period in a tertiary center in Tehran, Iran. We aimed to evaluate the results of biopsies as well as clinical and laboratory features of suspected GCA.

Although the overall mean age at biopsy in our patients was 65.5 years, we observed a significantly higher mean age in TAB-positive patients than that in TAB-negative ones (75.6 compared to 63.9 years). This finding reflects the increase in GCA incidence with age.

There is controversy regarding the impact of the length of biopsy on the results of TAB. Table 4 summarizes the characteristics of previously published studies about the association between TAB length and its diagnostic yield. Due to the probability of the presence of skip lesions, particularly in cases with insufficient specimen size, false-negative results (15–29%) may occur.^[12]^ Ashton-Key and Gallagher found 10–61% false negativity and 6% false positivity in TABs due to arteriosclerosis as a result of aging and not the inflammatory process.^[13]^

The ESR and CRP levels are biochemical markers that increase in GCA. We found a nonsignificant difference in the mean ESR between cases with a positive and those with a negative biopsy, but there was a statistically significant higher CRP level in the group of patients with a positive TAB than in the group with a negative TAB. However, no score has been considered for CRP according to the 2016 rACR criteria for the diagnosis of GCA.

The 1990 ACR diagnostic criteria for GCA consists of age at disease onset of 50 years or older, new headache, temporal artery abnormality, elevated ESR, and abnormal TAB. Omitting the biopsy item from the 1990 ACR score leaves only four criteria, which makes it difficult to justify not performing TAB for low- or high-risk patients. The applicability of the 1990 ACR criteria for the diagnosis of GCA according to its symptoms is another challenging issue. Murchison et al challenged the 1990 ACR criteria for the diagnosis of GCA.^[14]^ They found that nearly 25% of the patients with positive TABs did not fulfill the ACR criteria (probably because 20% of the GCA patients have occult GCA). In contrast, 28.3% of the patients met the criteria but were biopsy negative. Ing et al developed a new prediction model for diagnosing patients with suspected GCA.^[15]^ Their results are similar to ours in terms of more positive biopsy results in the elderly population as well as more jaw claudication, more ischemic optic neuropathy, and higher platelet levels, ESR, and CRP levels in the positive biopsy group than in the negative biopsy group. They found these variables significant predictors of positive TAB results. However, the 2016 rACR criteria has nine non-biopsy items, which could permit greater differentiation of patients with respect to the likelihood of a positive TAB result and GCA diagnosis. Based on the 2016 rACR criteria for the diagnosis of GCA, Sait et al proposed a simple management plan: (1) 2016 rACR scores ≤ 2 may not mandate a biopsy as these patients are very unlikely to have GCA; (2) patients with scores ≥ 5 will also not need TAB as they are likely to have GCA and should continue steroid therapy; and (3) a biopsy will be required in cases with scores of 3 and 4 because these patients have the most variability in TAB results.^[16]^ However, further studies involving multiple centers with firm inclusion criteria should be performed before an algorithm that avoids biopsy in GCA management is applied.

In our study, TAB-positive patients had a higher mean overall rACR score than the TAB-negative patients. One reason could be that TAB-positive patients with compatible pathology accumulate two more points in comparison with TAB-negative patients. Sait et al compared the functional utility of rACR criteria against the original ACR criteria and found that the more extensive rACR can serve as a more useful guide to reduce the number of unnecessary biopsies.^[16]^

In case of TAB-positive results in the first and second five years, the odds ratio was approximately 2.7 (comparing second five years to the first five years), even though the *P*-value was borderline. The increase in the specimen length can be due to human factors. TABs were performed by several different specialties in our center. Some TABs were carried out by neurosurgery and vascular surgery residents in the first five-year interval, but the Department of Ophthalmology was exclusively in charge of performing biopsies in the second five-year interval. The ophthalmology team was more experienced in the second five years than in the first; however, our results indicated that specimen length beyond a certain size did not influence the rate of positive results. The proficiency of the pathology team may also play an important role in analyzing specimens.

In recent years, other diagnostic modalities have gained popularity because of their high specificity, sensitivity, and ease of use. Color Doppler Ultrasound (CDUS) has been shown to have a specificity of 78–100% and a sensitivity of 55–100% for diagnosing GCA.^[17]^ Although extensive experience is needed for a proper diagnosis with CDUS, its high resolution of 0.1 mm and its noninvasiveness makes it a good choice as a diagnostic tool. With CDUS, not only the temporal artery can be visualized, but also other cranial arteries as well as axillary and subclavian arteries can be visualized for signs of vasculitis.^[18,19,20]^ High-resolution magnetic resonance imaging with magnetic resonance angiography (MRA) has been reported to have a pooled sensitivity of 73% and a specificity of 88%. This modality can show the temporal arteries and demonstrate mural edema if contrast is administered.^[21]^ Other modalities, including positron emission tomography (PET), computed tomography (CT), CT with angiography, and conventional MRA, lack sufficient spatial resolution to permit visualization of the temporal artery and hence are not the preferred modalities in GCA. Due to high fluorodeoxyglucose uptake in the brain, PET scanning of cranial arteries is considerably obscured.^[22]^

This study has several limitations. Because of its retrospective nature, we encountered a considerable amount of missing data that yielded a lower sample size than we originally anticipated. This might result in selection bias. Moreover, these results do not illustrate the whole population, as we included only patients from a tertiary referral center. Despite these limitations, this is the first study performed in Iran to evaluate the results of TAB and the clinical and laboratory characteristics of patients with suspected GCA.

In conclusion, we could not find an association between the analysis of TABs and the clinical evaluation of patients with GCA. Although TAB is still considered the gold standard test for GCA diagnosis, clinicians should consider both clinical and pathological data to manage difficult cases. This study shows that the majority of TABs are negative, and reducing the number of redundant biopsies is necessary to lower the workload of medical staff and the use of medical services.
